# Traffic symbol recognition modulates bodily actions

**DOI:** 10.1371/journal.pone.0214281

**Published:** 2019-03-25

**Authors:** Mayuko Iriguchi, Rumi Fujimura, Hiroki Koda, Nobuo Masataka

**Affiliations:** Primate Research Institute, Kyoto University, Inuyama, Japan; University of Toyama, JAPAN

## Abstract

Traffic signals, i.e., iconic symbols conveying traffic rules, generally represent spatial or movement meanings, e.g., “Stop”, “Go”, “Bend warning”, or “No entry”, and we visually perceive these symbols and produce appropriate bodily actions. The traffic signals are clearly thought to assist in producing bodily actions such as going forward or stopping, and the combination of symbolic recognition through visual perception and production of bodily actions could be one example of embodied cognition. However, to what extent our bodily actions are associated with the symbolic representations of commonly used traffic signals remains unknown. Here we experimentally investigated how traffic symbol recognition cognitively affects bodily action patterns, by employing a simple stimulus-response task for traffic sign recognition with a response of either sliding or pushing down on a joystick in a gamepad. We found that when operating the joystick, participants’ slide reaction in response to the “Go” traffic symbol was significantly faster than their push reaction, while their response time to the “Stop” signal showed no differences between sliding and pushing actions. These results suggested that there was a possible association between certain action patterns and traffic symbol recognition, and in particular the “Go” symbol was congruent with a sliding action as a bodily response. Our findings may thus reveal an example of embodied cognition in visual perception of traffic signals.

## Introduction

Embodied cognition has been discussed widely during the last few decades in several fields, such as psychology, linguistics and philosophy. The concepts of embodied cognition appear to oppose traditional views of human cognition, which posit that the mind as an information processor does not depend on the physical body [1,2]. These concepts focus on the embodiment of sensory and motor functions in cognition, and how the body modulates and shapes mental processing [3,4].

So far, many theoretical studies have discussed embodied cognition from various points of view. Cognitive linguistics theories, for example, argue that abstract concepts are metaphorically based on embodied knowledge [5,6]. Metaphors such as “good is up” and “life is a gamble” have underlying spatial orientation, and structured experiences and activities, respectively, and these metaphors fundamentally include aspects of how people think and understand [7]. Mental processing needs to be understood as involving the interaction between a physical body activities and its environment [8]. In other views, Barsalou [9,10] argued that perceptual symbols are often modal and represented in the same way as they are perceived, so he proposed his perceptual symbol system, which integrates traditional theories with theories of embodied cognition.

Empirically, embodied cognition has been tested to examine how the interactions between actions and cognition occurs. For instance, we use gestures to describe specific meanings such as shape, placement and motion in communication, and these gestural body movements are more frequently represented particularly when we express spatial concepts including directions, locations and motion in space (e.g., [11–13]). Some experiments showed that participants produced gestures more than twice as frequently when they spoke about spatial topics as when they spoke about verbal or non-spatial topics [11]. In a situation in which participants were prohibited from using gestures, their speech became less frequent, and when gestures and meanings of speech were not congruent, participants tended to produce more errors in conversation [14–17].

Finger counting, which clearly involves bodily actions, also conveys numerical concepts, and it supports people’s understanding of numbers, including arithmetical calculation [18,19]. In finger counting, the bodily actions are often spatially oriented relative to the horizontal (left/right) and vertical (top/bottom) axes, and these spatially dependent numerical axes are likely to be advantageous for the numerical recognitions (e.g., [20,21]). More fundamentally, visual perceptions are likely coupled with bodily actions, and humans process meanings of visual symbols or objects by responding with appropriate body movements [22–24]. A number of psychological experiments found that there were associations between visual stimuli such as images of objects (e.g., vegetables and clothes) or colour patches and bodily actions as responses to them, and that specific positions or symbolic concepts represented by visual stimuli could determine appropriate sensory modalities and promote bodily responses, as a congruent condition between visual stimuli and modality could produce faster reaction times or fewer errors in bodily responses, while incongruent conditions seemed to produce the opposite results [24–26]. In those experiments, participants answered concepts about an object (e.g., either the object was upright or inverted) or colours of a patch (e.g., either red or green) by pressing either a left or right button, and participants showed a significant delay of reaction times or more errors when concepts or colours of visual stimuli were not spatially congruent with the corresponding buttons to which they should react with their hands [23,24,27–29].

Pedestrian traffic signals we get used to in daily life have underlying symbolic meanings, “Go” and “Stop”, and we perceive these visual symbols and produce relevant bodily responses. We must perceive the meanings of the symbols and respond to them bodily as precisely and rapidly as possible: this means that the symbols’ designs should function to promote our appropriate bodily actions, and traffic symbols are an ideal set of visual symbols to empirically test regarding the embodied cognition. However, the possible cognitive-action interactions invoked by traffic signals still remain unclear.

Here we experimentally investigated the association between symbolic recognition and bodily action considering the concepts of embodied cognition, and examined the execution of action patterns in response to traffic signals by subjects using a joystick in a gamepad. A joystick is a common interface between a computer device and a player, and is used to simulate bodily actions, particularly in roleplay games in which a player becomes a character and produces relevant actions corresponding to recognition of symbols, conditions and environments in the game. We simply hypothesised that congruence/incongruence between traffic signals and the joystick reactions could influence the reaction time, based on concepts of embodied cognition.

## Materials and methods

### Ethics

All experiments were carried out in accordance with the Guidelines for Research in Human Participants, issued by the Human Research Ethics Committee of the Primate Research Institute, Kyoto University, and the experimental protocol was approved by the Committee (Permit No.2017-04). Before the experiments, we obtained written informed consent from all participants.

### Participants

Twenty-six adult participants (mean with standard deviation of age, 31.0 +/- 9.98 yrs, range 22–55 yrs) participated in the experiments. The participants included 10 males (age: 26.9 +/- 7.88 yrs, range: 22–47) and 16 females (age: 33.6 +/- 10.51, range: 23–55). All participants were Japanese and right handed, and participants did not show limited intellectual skills as tested by the Raven Coloured Progressive Matrices.

### Stimuli

**Fig 1:**
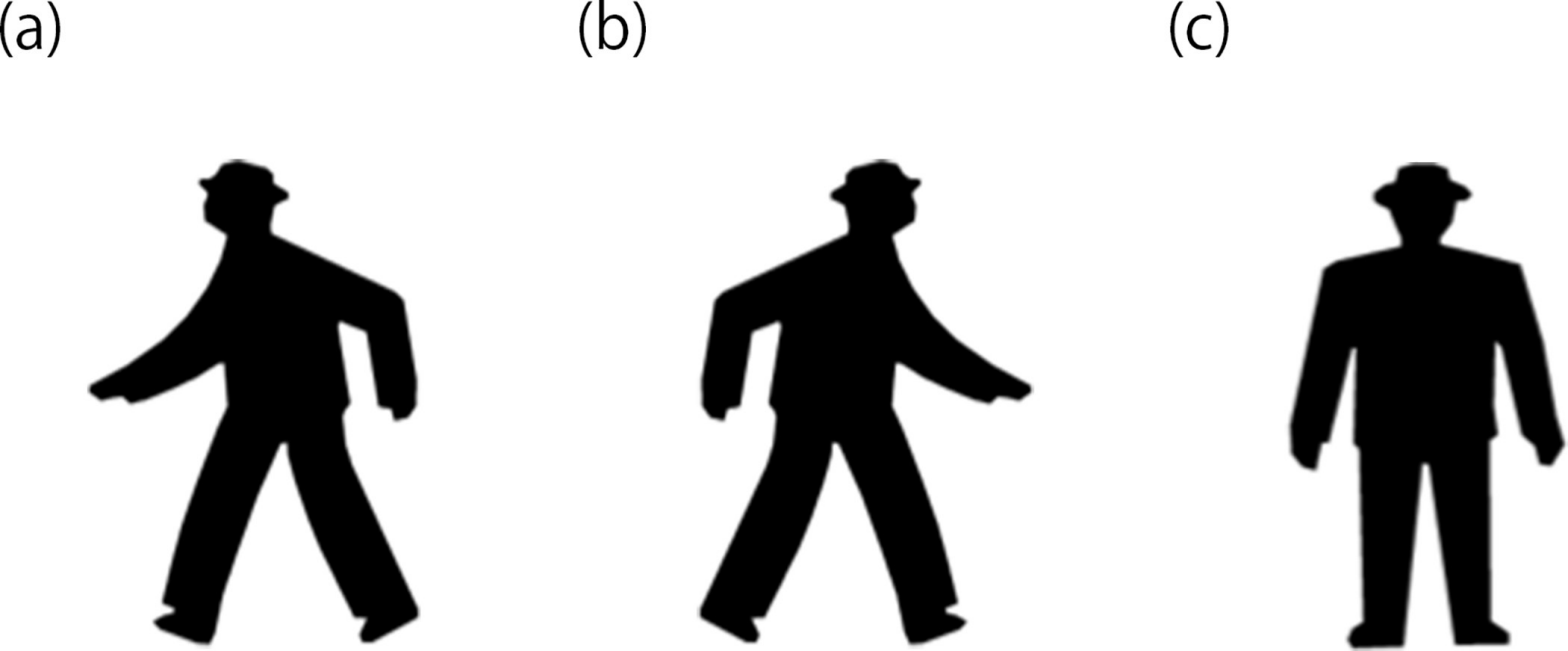
Type of stimuli. Stimuli in the experiments included three types: (a) Go-left, (b) Go-right and (c) Stop.

We used Japanese pedestrian traffic signals, which consisted of two types of symbol shapes, “a man walking from the right to the left side” as a “Go” symbol, or “a standing man” as a “Stop” symbol, with black-coloured conversion (Fig 1). In Japan, the Go signal always appears unidirectionally (walking from right to left), but we prepared Go signals using two directions, i.e., from right-to-left or left-to-right, in order to examine if an enhancement/interference effect of perception on horizontal action appears bidirectionally. We converted all stimuli to a size fitted in 250 x 250 pixels.

### Apparatus

All experiments were controlled using a custom-made program written using OpenSesame software ver. 3.1.6 (Mathôt, 2010–2016) on a laptop computer (HP Pavilion dv6, Tokyo, Japan) connected to a USB-gamepad (Elecom, JC-FU2912FBK, Japan). During the experiments, we recorded the reactions of participants on the gamepad in response to the traffic signal types “Go” or “Stop”.

### Procedure

**Fig 2:**
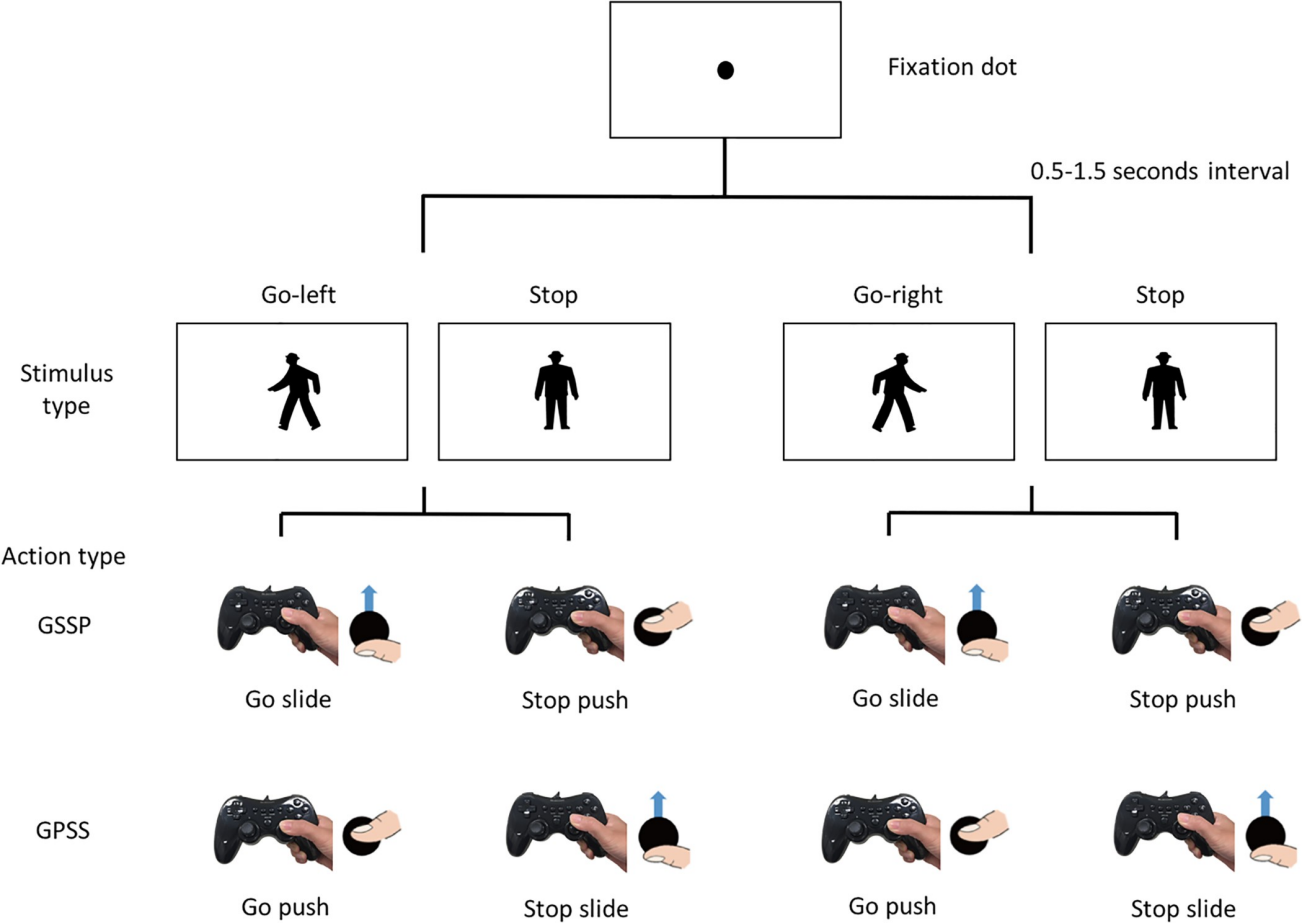
Experimental procedure. A fixation dot appeared, and after a 0.5–1.5 second interval, the fixation dot was replaced with one of two types of stimulus: either one of a pair of Go-left and Stop, or Go-right and Stop. Each stimulus of Go and Stop type appeared 18 times in random order, and participants responded to a stimulus type by gamepad actions: either the action type GSSP (Go Slide-Stop-Push) or GPSS (Go-Push-Stop-Slide). Each participant performed 144 trials: two types of stimulus (Go or Stop) x 18 times x two combinations (Go-left and Stop or Go-right and Stop) x two types of action (GSSP or GPSS).

Participants sat in front of the 16-inch screen of a laptop computer (resolutions: 1366 x 768 pixels), and held the gamepad with both hands. The screen was approximately 70 cm away from the participant, and the estimated visual angle of the stimuli was 20 degrees. First, in a single trial, a fixation dot with 8 pixels radius appeared at the centre of the screen. After 0.5–1.5 seconds, the fixation dot was replaced with either a “Go” or “Stop” stimulus at the centre of the screen. Participants were required to respond to either the “Go” or “Stop” stimulus by forward-sliding or pushing down, respectively, on the right joystick of the gamepad with the right hand. We examined two action conditions, i.e., “Go-Slide-Stop-Push (GSSP)” or “Go-Push-Stop-Slide (GPSS)” conditions. In the GSSP condition, the “Go” signal should be reacted to by forward-sliding of the joystick, and the “Stop” signal by pushing down on the stick (Fig 2). In contrast, in the GPSS condition, the “Go” signal should be reacted to by pushing down on the stick, and “Stop” by sliding forward (Fig 2). After the reactions, the next trial was started. First, the experiment included a practice phase with 16 trials (2 types of stimulus: Go or Stop x 8 times) for each of the “GSSP” condition and “GPSS” condition, and then the main phases. In the experiment, the main phases included 144 trials in total. These trials were divided into 2 action conditions, the “GSSP” and “GPSS” conditions, and the order of these conditions was counterbalanced for each participant. All participants carried out both conditions. Each condition consisted of 72 trials that also had 2 patterns, namely, combinations of the following stimuli: left-to-right-walking Go (Go-left stimulus) and Stop (Stop stimulus), and right-to-left-walking Go (Go-right stimulus) and Stop. In each pattern, Go (Go-left or Go-right) and Stop stimuli appeared 18 times in random order. During the experiments, the reaction times were always recorded.

### Analysis

The reaction times were analysed by analysis of variance tests (ANOVA) based on the linear mixed models in SPSS ver. 20. In the models, we set the action types (GSSP or GPSS) and stimulus shapes (Go or Stop) with their interaction effect terms (action type x stimulus shape) as the fixed main effect terms, and participant ID as a random effect term. When a significant interaction effect was observed, we examined the simple main effects as a post-hoc comparison. To examine the simple main effects in the mixed models of SPSS, we used the “Estimated Marginal Means” option with Bonferroni correction of the SPSS. We computed the 95% confidence intervals of the estimated marginal means between two comparison levels for each level of the other condition, and examined whether the two levels differed significantly.

## Results

**Fig 3:**
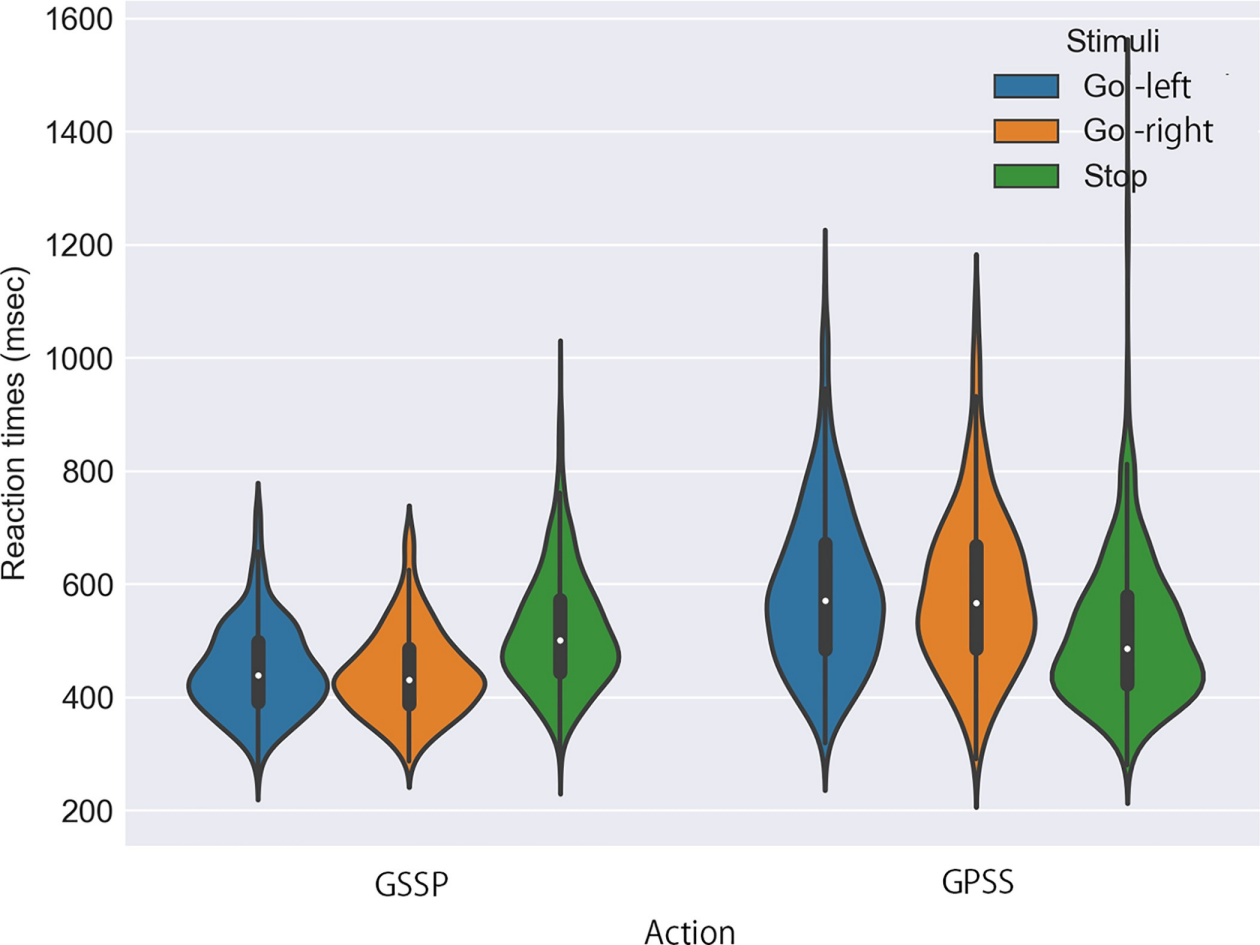
Average reaction time of participants (N = 26) according to the action performed on a gamepad joystick and stimulus shapes. The graph (left half) shows the average reaction time for different stimuli (Go-left, Go-right and Stop) with the action of sliding for Go and pushing for Stop (GSSP), and the graph (right half) shows the average reaction time for the same stimuli with the action of pushing for Go and sliding for Stop (GPSS).

When we conducted a two-way 2 x 3 ANOVA, we found a significant interaction effect between action type and stimulus shape (F_*2*, *120*.*937*_ = 46.469, *p* < 0.001, partial η^2^ = 0.090, Fig 3). Next, we performed post-hoc comparisons by computing the estimated marginal means. The reaction times for Go-Left or Go-Right stimuli were significantly shorter for the sliding reaction than for the pushing reaction, while those for the Stop stimulus did not significantly differ between the sliding and pushing reactions (Fig 3, Table 1 for the statistical details). Likewise, when comparing the reaction times for stimulus types for each action condition, we found that the reaction times for Stop stimuli were significantly longer than those for Go-Left or Go-Right stimuli in the case of GSSP, while we found the opposite in the case of GPSS (Fig 3, Table 1 for the statistical details). We also calculated the accuracy rates for each stimulus: Go-left, Go-right and Stop, in GSSP and GPSS, and found that they were 98.9% for Go-Left, 99.4% for Go-right and 97.3% for Stop in GSSP, and 97.9%, 95.1% and 98.8% in GPSS, respectively.

**Table 1:** Statistical results for post-hoc comparisons using the estimated marginal means.

Stimulus type	Action type		Mean difference	SE	p-value	95% confidence interval	
						lower bound	upper bound
Go-left	GSSP	GPSS	-140.272	7.836	.000	-155.635	-124.908
	GPSS	GSSP	140.272	7.836	.000	124.908	155.635
Go-right	GSSP	GPSS	-144.969	7.895	.000	-160.448	-129.489
	GPSS	GSSP	144.969	7.895	.000	129.489	160.448
Stop	GSSP	GPSS	3.318	5.582	.552	-7.626	14.262
	GPSS	GSSP	-3.318	5.582	.552	-14.262	7.626

Post-hoc comparisons between stimulus type (Go-left, Go-right or Stop) and action type (GSSP or GPSS).

## Discussion

The experiment reported here clearly showed faster reaction, particularly for the “Go” stimulus, in the GSSP condition, which would be assumed to be a “congruent” condition, and slower reaction for the “Go” stimulus in the GPSP condition, which would be assumed to be an “incongruent” condition. That is, in particular, the participants easily slid the sticks for “Go”. In contrast, they had difficulty in operating the stick in the reverse way: pushing down for the “Go” decision. For “Stop” stimulus, a delay or shortening of reaction time was not observed in either the GSSP or GPSP condition. These results suggested that cognitive-motor engagement could be observed when recognising traffic signs, particularly for motions such as pedestrian walking, providing evidence of embodied cognition.

Traffic signals are familiar icons for the representation of traffic rules, which historically started in London in the late 19^th^ century as actual physical movements of police officers’ arms and hands and blowing of whistles [30], conveying the rules in order to avoid serious traffic accidents. To achieve their purpose, the traffic signals require us to quickly perceive their symbolic meanings and react with appropriate body operations, and thus traffic signals should be designed to be as quickly and easily understandable as possible. In fact, misunderstanding of the signal meaning often leads to the critical traffic accidents [31,32], suggesting the importance of the signal designs. Recently, the influence of the signal designs for the symbol recognitions has been reported. The memory task of traffic signals revealed that some symbolic features enhances their memorizations of signal meaning, e.g., an advantage of signals made from Chinese characters for Chinese people [33–35]. Likewise, signal designs (e.g., arrow direction) influence their driving abilities when the drivers recognise the signal meaning [36]. Some recent studies revealed strong relationships between recognition of traffic signals and bodily actions. For example, traffic signals representing obligatory and prohibited actions effectively influence decision making and responses in appropriate mental processing of the signals’ meanings [37–39]. When recognizing traffic signs including the direction (represented by an arrow) of an airport (represented by an airplane), participants could also most rapidly and precisely detect the meaning of directionally congruent signs of the airplane and arrow [36]. How we visually perceive and process the meanings of traffic signals in our cognition possibly affects actual bodily actions.

A gamepad or similar devices are familiar tools for computer games, and these are also used in many experiments such as driving simulation in order to examine drivers’ behaviour [36,40,41]. In computer games, a joystick in a gamepad is commonly and easily used for everyone in Japan. A player act as a character on the screen and move the body of character by a joystick, and forward sliding is often associated with forward moving of character. Similarly, in experiments, a gamepad or similar devices were used in order to examine traffic symbol recognition and drivers’ behaviour. Recognition of airport direction signs with an arrow and airplane symbols were examined using a gamepad action, and this study revealed that congruence between signs and bodily actions possibly improves traffic signage [36].

As shown in our study, congruence between perception of symbols and body action is fundamental for producing responses. In particular, recognition of a visual “Go” symbol was more likely to cause a faster reaction time of a sliding action than of a pushing down action. This is consistent with various congruences regarding other actions, e.g., gestures or finger counting. Gestures convey specific meanings in conversation, and those meanings are often associated with direction, location and motion involving space, and body movements used as gestures and the meanings should be matched to facilitate understanding and communication of correct meanings [11–13]. Finger counting clearly shows the magnitude of number-associated space (the SNARC effect), and these concepts underlie the bodily action of finger counting, combined with eye gaze or changing body directions during counting numbers [42–44]. Bodily movements such as gestures and finger counting enable us to transmit information more effectively and enhance understanding when meanings and bodily movements are congruent in our mind. These effects of the congruency between symbolic meanings and bodily actions have been observed in studies of both gestures and finger counting, and incongruent conditions can cause errors and decreases of fluency and understanding of meanings [14–18,45]. The results of our study indicated that the symbolic meaning of “Go” may be more associated with sliding action than with pushing action, and “Stop” may possibly be more associated with pushing action than with sliding action, and whether there was congruency or incongruency between the recognition of a symbol and bodily actions could affect reaction times.

As with gestures and finger counting, visual perception such as perception of symbols is strongly associated with bodily actions, and incongruence between symbolic meanings and perceptions of symbols could cause negative effects on responses, such as errors or delays of reaction time [24–26]. We observed that participants responded to a “Go” stimulus faster by a sliding action than by a pushing action, and “Go” symbol might be congruent with a sliding action. At this stage, however, we could not conclude whether the symbols could either enhance the bodily action of sliding a joystick, or inhibit that of pushing it in this experimental study, or whether our findings could have occurred due to a combination of both enhancement and inhibition, because any neutral conditions were lacking in our experiments. Some reported studies examined whether either an enhancement effect or inhibition effect impacted the variation of reaction time or accuracy of performance in experimental tasks such as word-translation and spatial orienting paradigm, and those studies examined which effect, enhancement or inhibition, could determine subjects’ responses [46–50]. For instance, in spatial attention tasks, a cue appeared on the PC screen to fixate a participant’s eye location in the centre once before the target stimulus appeared, and then a participant moved their eyes to the target [50]. In these tasks, they used the neutral condition stimuli to separate the stimulus influence of “enhancement” from “inhibition.” Thus, participants in our experiments might have responded to symbols by bodily actions according to either enhancement or inhibition effects, or even both. In the near further, we should test which effects have more influence on bodily responses, by applying a neutral condition to enable a deep understanding of stimulus-action interaction.

## Conclusions

Our results clearly suggested a common embodied cognition in visual perception shown as a cognition-action association between symbolic recognition of traffic signals, “Go” and “Stop”, and bodily actions of sliding and pushing of a joystick. Congruence between symbolic recognition and bodily action may enhance participants’ responses, and incongruence may produce the opposite effects. This is a fundamental idea for understanding embodied cognitive features of recognition of symbolic meanings and for future practices of traffic signal design.

## Supporting information

S1 Dataset Data of reaction times of participants. (CSV)
